# Non-speculum sampling approaches for cervical screening in older women: randomised controlled trial

**DOI:** 10.3399/BJGP.2021.0350

**Published:** 2021-12-31

**Authors:** Rebecca Landy, Tony Hollingworth, Jo Waller, Laura AV Marlow, Jane Rigney, Thomas Round, Peter D Sasieni, Anita WW Lim

**Affiliations:** Division of Cancer Epidemiology and Genetics, National Cancer Institute, National Institutes of Health, Department of Health and Human Services, Bethesda, Maryland, US.; Whipps Cross University Hospital, Barts Health NHS Trust, London.; Comprehensive Cancer Centre, School of Cancer and Pharmaceutical Sciences, Faculty of Life Sciences and Medicine, King’s College London, London.; Comprehensive Cancer Centre, School of Cancer and Pharmaceutical Sciences, Faculty of Life Sciences and Medicine, King’s College London, London.; Comprehensive Cancer Centre, School of Cancer and Pharmaceutical Sciences, Faculty of Life Sciences and Medicine, King’s College London, London.; School of Population Health and Environmental Sciences, King’s College London, London and National Cancer Analysis and Registration Service, Public Health England.; Comprehensive Cancer Centre, School of Cancer and Pharmaceutical Sciences, Faculty of Life Sciences and Medicine, King’s College London, London.; Comprehensive Cancer Centre, School of Cancer and Pharmaceutical Sciences, Faculty of Life Sciences and Medicine, King’s College London, London.

**Keywords:** Cervical intraepithelial neoplasia, early detection of cancer, general practice, human papillomavirus DNA tests, older women, self-sampling

## Abstract

**Background:**

Cervical cancer disproportionately affects women ≥65 years, especially those not screened regularly. Speculum use is a key barrier.

**Aim:**

To assess if offering non-speculum clinician-taken sampling and self-sampling increases uptake for lapsed attenders aged 50–64 years.

**Design and setting:**

Pragmatic randomised control trial conducted at 10 general practices in East London, UK.

**Method:**

Participants were 784 women aged 50–64 years, last screened 6–15 years before randomisation. Intervention participants received a letter offering the choice of non-speculum clinician- or self-sampling. Control participants received usual care. The main outcome measure was uptake within 4 months.

**Results:**

Screening uptake 4 months after randomisation was significantly higher in the intervention arm: 20.4% (*n* = 80/393) versus 4.9% in the control arm (*n* = 19/391, absolute difference 15.5%, 95% confidence interval [CI] = 11.0% to 20.0%, *P*<0.001). This was maintained at 12 months: intervention 30.5% (*n* = 120/393) versus control 13.6% (*n* = 53/391) (absolute difference 17.0%, 95% CI = 11.3% to 22.7%, *P*<0.001). Conventional screening attendance within 12 months was very similar for both intervention 12.7% (*n* = 50/393) and control 13.6% (*n* = 53/391) arms. Ethnic differences were seen in screening modality preference. More White women opted for self-sampling (50.7%, *n* = 38/75), whereas most Asian and Black women and those from other ethnic backgrounds opted for conventional screening.

**Conclusion:**

Offering non-speculum clinician-taken sampling and self-sampling substantially increases uptake in older lapsed attendee women. Non-speculum clinician sampling appeals to women who dislike the speculum but still prefer a clinician to take their sample. Providing a choice of screening modality may be important for optimising cervical screening uptake.

## INTRODUCTION

High cervical cancer mortality rates in older women have been observed in several countries.^1^^–^^3^ In the UK, women aged ≥65 years account for around half of cervical cancer deaths^4^ and 20% of new cases.^5^ Most of these arise in women who were screened inadequately when aged 50–64 years,^6^ ages at which screening coverage declines.^7^ With the current projections for increases in life expectancy,^8^ the number of cervical cancers in women aged >65 years is expected to rise. The negative impact of the COVID-19 pandemic on screening could further compound the issue.^9^^–^^10^ The speculum examination is a well-known barrier to cervical screening and can become particularly uncomfortable for older women because of vaginal atrophy, increasing body mass index and musculoskeletal problems.^11^^–^^12^ Studies show that older women find cervical screening/insertion of the speculum more painful with age and the menopause.^13^^–^^14^

An obvious solution is to offer HPV (human papillomavirus) testing on self-collected samples.^15^ Self-sampling enables women to collect their own sample for cervical screening without a speculum using a vaginal swab or brush. A drawback is the consistent finding that women worry about not self-sampling correctly.^16^^–^^19^ Offering a clinician-taken sample for HPV testing without a speculum (that is ‘non-speculum HPV testing’) is another option. Women would have the reassurance of a clinician-taken sample without the discomfort of speculum insertion. This approach could be particularly appealing to older lapsed attendees who have found screening increasingly uncomfortable with age but lack confidence in self-sampling. Women who have never attended screening by age 50 years are more entrenched in their decision to not attend^12^ and therefore less likely to respond to such interventions, regardless of what test is offered.

Previously the current author group has found that non-speculum clinician sampling was an appealing option for older women, particularly for those who may have been put off screening by the speculum examination.^11^ The aims of the present study were to assess the increase in screening uptake associated with offering lapsed attenders aged 50–64 years the option of non-speculum clinician-collected sampling or self-sampling, and the feasibility and acceptability of non-speculum clinician sampling. In addition, differences in uptake by ethnic background were explored.

**Table:** How this fits in

Women aged ≥65 years are at a disproportionately higher risk of cervical cancer and dying from it if they are underscreened. Speculum use is a major barrier to cervical screening and can become more uncomfortable with ageing and the menopause. Although self-sampling has been hailed as a game-changer for cervical screening, it does not appeal to all women. This study showed that offering a choice of non-speculum clinician-taken sampling or self-sampling substantially increased cervical screening uptake in older lapsed attendees across all ethnicities, an approach that could be easily implemented into existing practice in primary care.

## METHOD

Eighteen general practices in East London (UK) were invited to take part in this pragmatic, randomised controlled trial (ISRCTN16007231). Of these, 10 participated, all from the boroughs of Tower Hamlets or City and Hackney. Both boroughs have an ethnically diverse population with 55% and 45% from Black, Asian and other minority ethnic backgrounds, respectively.^20^ In England, women aged 50–64 years are sent 5-yearly screening invitations with reminder letters at 18 weeks. Individual GP practices may also provide additional reminders via telephone, text message, or letter. Women book their own appointments, which are conducted in GP primary care.

Eligible women were identified using the GP electronic patient record system EMIS Web between August 2018 and November 2018. These comprised women aged 50–64 years on the search date, who were at least 12 months overdue but attended at least one cervical screening in the previous 15 years. Randomisation was conducted before consent using Zelen’s design^21^ to allow unbiased assessment of the intervention.

In total, 809 women were randomised 1:1 to either the intervention or control arm within each practice. Randomisation was performed separately by each GP practice (on the same day as the EMIS search); to ensure equal numbers from each GP practice were assigned to each study arm (details in Supplementary Appendix S1). Invitation letters were sent to women randomised to the intervention on the same day as randomisation (or next working day). Follow-up data were obtained until November 2019.

Women in the intervention arm were sent an English-language mailout (see Supplementary Appendix S2) including an invitation letter, a study information leaflet, an HPV information sheet, and a self-sampling kit postal order form with a prepaid return envelope. The invitation letter offered women the option of:

- booking an appointment at their GP practice for a clinician-taken sample without using a speculum (a non-speculum sample), or
- ordering a self-sampling kit (using the postal order form or telephone).

The letter also reminded women they could book in for a standard cervical screening appointment.

Difficulty in booking appointments is a known screening barrier,^22^ therefore GP practices were asked to provide additional routes to make it easier for women to book screening appointments (see Supplementary Appendix S1). Women randomised to the control arm received usual care, that is sent invitation letters for cervical screening every 5 years until age 64 years and remain eligible for screening in between invitations if they are overdue.

Non-speculum samples were taken at the GP practice by the usual cervical screening sample-takers. Sample-takers were provided with written and pictorial instructions for collecting the sample (Supplementary Appendix S3).

Women who ordered self-sampling kits had a kit posted to their home address. Self-sampling kits included a flocked swab (FLOQSwab 552C™, Copan Italia, Brescia, Italy), a laboratory request consent form, a freepost envelope pre-addressed to the testing laboratory, written and pictorial instructions detailing how to collect a self-sample, a study information leaflet, an HPV information sheet, and a questionnaire (Supplementary Appendix S4).

Study samples (non-speculum and self-samples) were tested for the presence of HPV DNA. Conventional screening samples were tested as per the national programme at the time (liquid-based cytology with HPV triage). HPV test results were posted to women, copied to their GP practice. HPV positive results letters advised women to book a conventional (speculum) follow-up test. Women were managed according to the result of the conventional test under the national cervical screening programme.

A questionnaire (Supplementary Appendix S5) was included in non-speculum and self-sampling kits for women to complete after sample-taking, eliciting information about women’s experience of the test (using four-point Likert scales), previous barriers to screening, and future screening preferences.

All samples were analysed within 7 days of receipt by the Cytology Department at Barts Health NHS Trust, London, UK. HPV testing was performed using Cobas® 4800 HPV Test (Roche Diagnostics GmBH). For details on the laboratory analyses, see Supplementary Appendix S1.

### Statistical analysis

Electronic GP records provided data on each woman’s age, ethnicity, cervical screening attendance, cervical screening results, and time since the last recorded screen. The laboratory provided data on study sample HPV results, cytology, and colposcopy data.

Statistical analyses were pre-specified in the protocol and described in the statistical analysis plan; additional analyses are noted as such. The primary outcome was the proportion of women with any form of cervical screening within 4 months by study arm. The study was powered to detect a difference in screening uptake of 6% in controls versus 13% in the intervention arm at 4 months: a sample size of 367 per arm would give 90% power with a two-sided alpha of 0.05. It was assumed that uptake in the control arm would be 4–8%, and a sample of 800 participants would give between 75% and 93% power under a range of scenarios. Secondary analyses considered:

- screening within 12 months;
- differences in uptake by age, ethnicity, and time since the last screen; and
- perceptions of the sampling approaches.

Evaluating uptake within 12 months enabled us to assess whether any increased uptake seen at 4 months was maintained, rather than being a ‘nudge’ effect prompting women who would have attended anyway, to be screened earlier.

The proportion in each study arm who had any form of screening was reported stratified by age at randomisation, ethnic background, and time since last screen (‘late’, 6–>10 years; ‘very late’, ≥10–15 years). χ^2^-squared tests (or Fisher’s exact tests, if there were <5 women expected in any cell) were performed to assess differences in the type of screening test selected by stratification variables in the intervention arm. Logistic regression analyses investigated potential interactions between study arm and each of a) age, b) ethnicity, and c) time since the last screening, with the outcome of screening uptake (not pre-specified in the protocol). A Kaplan–Meier plot was produced, showing the time of screening for the control arm versus the intervention arm for: a) conventional (speculum) screening, b) conventional (speculum) screening or self-sampling, and c) any form of screening.

For questionnaire data, differences between attitude items towards non-speculum sampling and self-sampling were dichotomised and explored using χ^2^ tests (or Fisher’s exact tests if appropriate).

## RESULTS

A total of 809 women were randomised in the study (intervention *n* = 404, control *n* = 405). Of these, 16 were found to be ineligible because of inaccurate GP screening records. A further nine were excluded as information on their screening attendance during the trial period was not available (they were not in the GP record system at final data collection having presumably left the GP practice). Therefore, 393 eligible women were in the intervention arm and 391 in the control arm.

**Figure 1.**
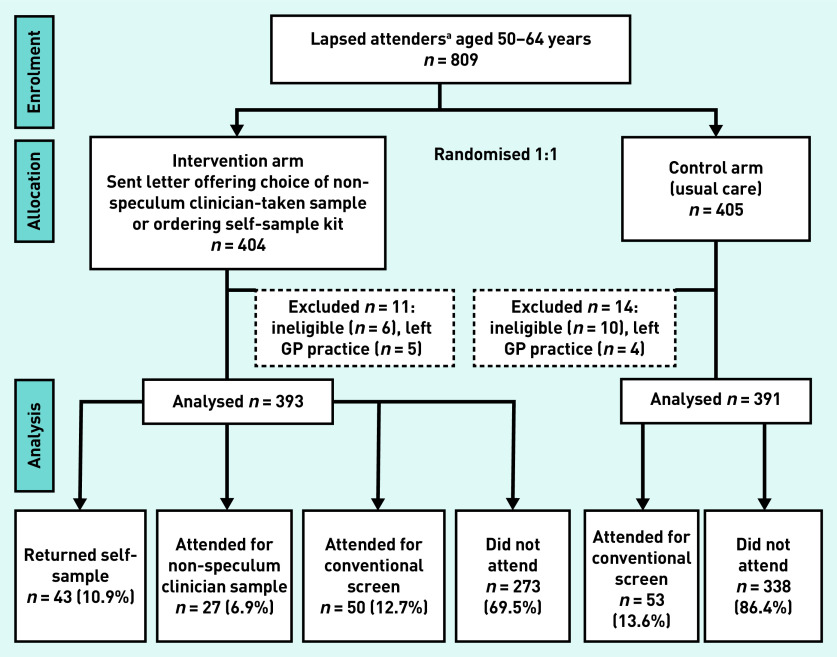
***Trial flow diagram***. *^a^* ***Lapsed attenders were defined as women with at least 12 months overdue screening but who had attended at least once in the previous 15 years according to GP records.***

Table 1 shows demographic characteristics and Figure 1 shows the study flowchart. In total 43% were from Black, Asian, and other minority ethnic backgrounds. The number of women from each GP practice ranged from 21 to 172. A summary of the characteristics of the participating GP practices is provided in Supplementary Table S1.

**Table 1.** Demographic characteristics of trial participants, by intervention arm

**Characteristic**	**Intervention**		**Control**	
	
	***n***	**%**	***n***	**%**
**Total**	393	100	391	100

**Age, years**				
50–54	127	32.3	135	34.5
55–59	155	39.4	131	33.5
60–64	111	28.2	125	32.0

**Ethnic background**				
White	229	58.3	218	55.8
Black	69	17.6	67	17.1
Asian	56	14.2	67	17.1
Mixed/other/unknown	39	9.9	39	10.0

**Time since their last screening test**				
Late (6–<10 years)	272	69.2	264	67.5
Very late (10–15 years)	121	30.8	127	32.5

**GP practice**				
1	16	4.1	15	3.8
2	15	3.8	16	4.1
3	58	14.8	54	13.8
4	50	12.7	49	12.5
5	50	12.7	47	12.0
6	36	9.2	39	10.0
7	8	2.0	13	3.3
8	50	12.7	51	13.0
9	87	22.1	85	21.7
10	23	5.9	22	5.6

Uptake 4 months after randomisation was significantly higher in the intervention arm versus the control arm: 20.4% (*n* = 80/393) versus 4.9% (*n* = 19/391, Table 2); absolute difference 15.5% (95% confidence interval [CI] = 11.0% to 20.0%, *P*<0.001). This difference was maintained at 12 months; 30.5% (*n* = 120/393) versus 13.6% (*n* = 53/391) in the intervention arm versus the control group; absolute difference 17.0% (95% CI = 11.3% to 22.7%, *P*<0.001). Conventional screening uptake within 12 months was very similar in the two arms, intervention 12.7% (*n* = 50/393) and control 13.6% (*n* = 53/391). Of those screened in the intervention arm, 22.5% (*n* = 27/120) had a non-speculum clinician sample, 35.8% (*n* = 43/120) had a self-sample, and 41.7% (*n* = 50/120) had a conventional (speculum) sample (Table 3).

**Table 2.** Percentage of women screened within 4 months and 12 months by intervention arm, by age, ethnicity, time since last screen and GP practice

**Characteristic**	**Screened, % (*n* screened/*n* eligible)**			

	**Within 4 months**		**Within 12 months**	
	
	**Intervention**	**Control**	**Intervention**	**Control**
**Total**	20.4 (80/393)	4.9 (19/391)	30.5 (120/393)	13.6 (53/391)

**Age, years**				
50–54	19.7 (25/127)	5.9 (8/135)	29.1 (37/127)	16.3 (22/135)
55–59	21.3 (33/155)	5.3 (7/131)	32.9 (51/155)	12.2 (16/131)
60–64	19.8 (22/111)	3.2 (4/125)	28.8 (32/111)	12.0 (15/125)

**Ethnic background**				
White	23.1 (53/229)	2.8 (6/218)	32.3 (74/229)	11.0 (24/218)
Black	20.3 (14/69)	4.5 (3/67)	29.0 (20/69)	17.9 (12/67)
Asian	16.1 (9/56)	11.9 (8/67)	26.8 (15/56)	19.4 (13/67)
Mixed/other/unknown	10.3 (4/39)	5.1 (2/39)	23.1 (9/39)	10.3 (4/39)

**Time since the last screening test**				
Late (6–<10 years)	23.9 (65/272)	6.1 (16/264)	36.0 (98/272)	15.9 (42/264)
Very late (≥10–15 years)	12.4 (15/121)	2.4 (3/127)	18.2 (22/121)	8.7 (11/127)

**GP practice**				
1	6.3 (1/16)	0.0 (0/15)	25.0 (4/16)	20.0 (3/15)
2	33.3 (5/15)	6.3 (1/16)	40.0 (6/15)	12.5 (2/16)
3	19.0 (11/58)	1.9 (1/54)	24.1 (14/58)	7.4 (4/54)
4	16.0 (8/50)	4.1 (2/49)	28.0 (14/50)	8.2 (4/49)
5	26.0 (13/50)	8.5 (4/47)	30.0 (15/50)	12.8 (6/47)
6	5.6 (2/36)	10.3 (4/39)	22.2 (8/36)	17.9 (7/39)
7	0.0 (0/8)	0.0 (0/13)	12.5 (1/8)	15.4 (2/13)
8	22.0 (11/50)	2.0 (1/51)	30.0 (15/50)	7.8 (4/51)
9	29.9 (26/87)	5.9 (5/85)	44.8 (39/87)	21.2 (18/85)
10	13.0 (3/23)	4.5 (1/22)	17.4 (4/23)	13.6 (3/22)

**Table 3.** Screening test selected among women who were screened within 4 months and 12 months by age, ethnic background, and time since last screening test^a^

**Characteristic (**χ**^2^)^b^**	**Women in the intervention arm who were screened within 4 months, % (*n*)**					**Women in the intervention arm who were screened within 12 months, % (*n*)**				***P*-value**
	
	**Non-speculum**	**Self-sample**	**Speculum**	**Total, *n***	***P*-value (**χ^2^**)^b^**	**Non-speculum**	**Self-sample**	**Speculum**	**Total, *n***	
**Age, years**					0.066					0.164
50–54	28.0 (7)	40.0 (10)	32.0 (8)	25		24.3 (9)	27.0 (10)	48.6 (18)	37	
55–59	18.2 (6)	48.5 (16)	33.3 (11)	33		15.7 (8)	37.3 (19)	47.1 (24)	51	
60–64	45.5 (10)	50.0 (11)	4.5 (1)	22		31.3 (10)	43.8 (14)	25.0 (8)	32	

**Ethnic background**					<0.001					<0.001
White	24.5 (13)	62.3 (33)	13.2 (7)	53		21.3 (16)	50.7 (38)	28.0 (21)	75	
Black	28.6 (4)	7.1 (1)	64.3 (9)	14		19.0 (4)	9.5 (2)	71.4 (15)	21	
Asian	66.7 (6)	0.0 (0)	33.3 (3)	9		46.7 (7)	0.0 (0)	53.3 (8)	15	
Mixed/other/unknown	0.0 (0)	75.0 (3)	25.0 (1)	4		0.0 (0)	33.3 (3)	66.7 (6)	9	

**Time since the last screening test**					0.185					0.241
Late (6– <10 years)	27.7 (18)	43.1 (28)	29.2 (19)	65		22.4 (22)	32.7 (32)	44.9 (44)	98	
Very late (≥10–15 years)	33.3 (5)	60.0 (9)	6.7 (1)	15		22.7 (5)	50 (11)	27.3 (6)	22	

**Total**	28.8 (23)	46.3 (37)	25.0 (20)	80		22.5 (27)	35.8 (43)	41.7 (50)	120	

a *Percentages are row percentages.*

b *Fisher’s exact test used for ethnic background.*

For the intervention arm, women who were ‘late’ were more likely to be screened within 4 months than women who were ‘very late’ (that is 6–<10 years versus 10–15 years since their last screening test, respectively) (23.9% versus 12.4%, *P* = 0.016) (Table 2). This remained true at 12 months (36.0% versus 18.2%, *P*<0.001). No statistically significant differences in uptake by age, or ethnicity were observed in the intervention arm (Table 2). However, a trend for decreasing uptake with increasing age was observed in the control arm but not the intervention arm.

Selection of screening test differed by ethnicity in the intervention arm (*P*<0.001). Within 12 months, half the screened women from White backgrounds self-sampled (50.7%, *n* = 38/75), whereas the majority of women from Asian (53.3%, *n* = 8/15), Black (71.4%, *n* = 15/21), and Mixed/other/unknown backgrounds (66.7%, *n* = 6/9) attended conventional (speculum) screening (Table 3).

Differences by age (*P* = 0.066 [within 4 months] and *P* = 0.164 [within 12 months]) and time since the last screen (*P* = 0.185 [within 4 months] and *P* = 0.241 [within 12 months]) were not statistically significant, although an increasing proportion of screened women had a conventional (speculum) sample with increasing age.

**Figure 2.**
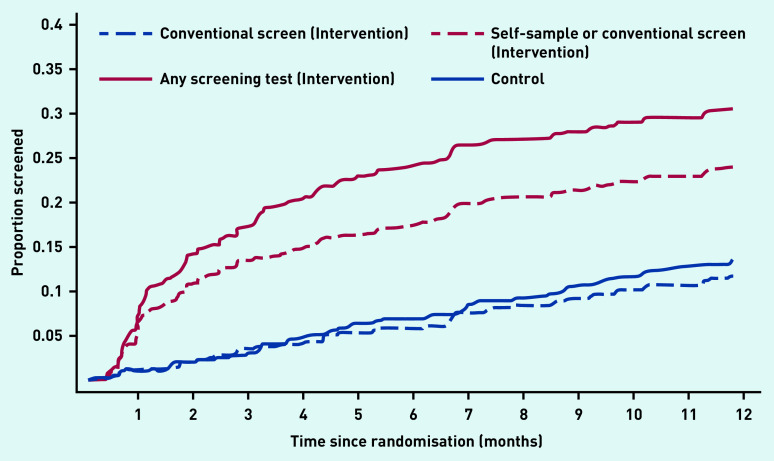
*Kaplan–Meier plot showing time to screening for the intervention and control arms for the various sampling approaches. The distance between ‘Conventional screen (intervention)’ (dashed blue line) and ‘Any screening test (Intervention)’ (solid red line) is the additional uplift in screening from non-speculum clinician sampling and self-sampling. The difference between the ‘Self-sample or conventional screen (intervention)’ (red dashed line) and ‘Any screening test (intervention)’ (red solid line) represents the number of women with a non-speculum clinician-taken sample. All screening in the control arm is conventional (speculum) screening as this is the only screening method currently available in the England national screening programme.*

Figure 2 shows the Kaplan–Meier plot for screening attendance up to 12 months. The pattern of screening uptake for conventional screening (clinician-sampled speculum) was very similar for both study arms. Self-sampling was most common in the first month. All non-speculum clinician samples were collected within 5 months.

Of the 393 women in the intervention arm, 63 (16.0%) ordered a self-sampling kit, and of these 43 (68.3%) returned a sample. Information on the number of women who booked versus attended a non-speculum clinician appointment was not available.

The vast majority (94.3%, 66/70) of women who returned a study sample tested HPV negative. Four women tested HPV positive: two non-speculum clinician samples, and two self-samples; all attended appropriate follow-up. The two non-speculum screen positives had abnormal cytology (one mild dyskaryosis, one moderate dyskaryosis); both attended colposcopy and had normal histology on biopsy. Both self-sample screen positives had negative cytology.

The questionnaire response rate was 85.7% (60/70) and was lower for non-speculum clinician sampling (66.7% [18/27] versus self-sampling 97.7% [42/43]). Both approaches scored similarly in measures of acceptability and confidence in doing the test properly (Table 4). By contrast, a higher proportion of self-samplers were ‘not at all’ or ‘not very’ confident in the test accuracy (64.3% versus 23.5% in the non-speculum group, *P* = 0.009). More women who had the non-speculum test experienced embarrassment (27.8% versus 4.8% in the self-sample group; *P* = 0.021) and believed it was important to have a clinician take the sample (88.9% [16/18] versus 26.2% [11/42], respectively *P*<0.001). A high proportion in both groups (72.2% [13/18] non-speculum, 88.1% [37/42] self-sampling) ‘agreed’ or ‘strongly agreed’ that it was important to have a choice of tests (data not shown). Future screening preferences aligned with the sampling option chosen. Small numbers limited the ability to assess previous barriers to screening. Nevertheless, both groups endorsed similar barriers, although a higher proportion of self-samplers endorsed embarrassment and practical barriers to screening.

**Table 4.** Perceptions of non-speculum clinician sampling versus self-sampling and previous barriers to screening

	**Non-speculum (*N*= 18)^a^ *n* (%)**	**Self-sample (*N*= 42)^a^ *n* (%)**	***P*-value (**χ^2^**)^b^**
**Overall experience of test**			0.735
Excellent/good	15 (83.3)	37 (88.1)	
Fair/poor	3 (16.7)	5 (11.9)	

**Discomfort**			0.775
None	11 (61.1)	24 (57.1)	
Mild/quite a lot/severe	7 (38.9)	18 (42.9)	

**Unpleasantness**			0.07
Not at all	11 (61.1)	34 (82.9)	
Mildly/fairly/very	7 (38.9)	7 (17.1)	

**Embarrassment**			0.021
Not at all	13 (72.2)	40 (95.2)	
Mildly/fairly/very	5 (27.8)	2 (4.8)	

**Anxiety**			0.952
Not at all	12 (66.7)	27 (65.9)	
Slightly/fairly/very	6 (33.3)	14 (34.2)	

**Confidence test done properly**			1.0
Not at all/not very	1 (5.9)	2 (4.9)	
Fairly/very	16 (94.1)	39 (95.1)	

**Confidence in test accuracy**			0.009
Not at all/not very	4 (23.5)	27 (64.3)	
Fairly/very	13 (76.5)	15 (35.7)	

**Future preference**			<0.001
Non-speculum	12 (70.6)	1 (2.4)	
Self-sample	4 (23.5)	38 (90.5)	
Speculum	1 (5.9)	0 (0)	
No preference	0 (0)	3 (7.1)	

**Previous barriers to screening^c^**			
Forgotten	4 (22.2)	7 (16.7)	0.719
More important things to worry about	0	5 (11.9)	0.309
Too busy	2 (11.1)	9 (21.4)	0.478
Not sexually active	4 (22.2)	11 (26.2)	1.0
Pain	10 (56.6)	23 (54.8)	0.955
Same partner long time	0	3 (7.1)	0.547
Too embarrassed	0	8 (19.1)	0.091
Frightened	2 (11.1)	1 (2.4)	0.212
Bad experience	3 (16.7)	13 (31.0)	0.346
Decided not worth going for screening	1 (5.6)	5 (11.9)	0.658

**Important to have a clinician take the sample**			
Not important/somewhat important	2 (11.1)	31 (73.8)	<0.001
Fairly important/very important	16 (88.9)	11 (26.2)	

a *Please note there are missing data for some questions.*

b *Fisher’s exact test was used for all data except experience, discomfort, unpleasantness, and anxiety.*

c *Participants could endorse more than one barrier.*

## DISCUSSION

### Summary

Offering non-speculum and self-sampling significantly increased screening uptake among older women who were lapsed attenders for screening. The fact that uplift remained at 12 months suggests that these women would not have otherwise attended. Encouragingly, increased uptake was observed across all ethnic backgrounds, age groups, and screening histories. These findings provide further evidence that offering women a choice is important and will be conducive to higher screening uptake. Although more women opted for self-sampling than non-speculum clinician-taken sampling, the fact that a substantial proportion chose the latter suggests that it appeals to the older lapsed attender population and could increase uptake beyond offering self-sampling alone.

### Strengths and limitations

To the authors’ knowledge, offering HPV testing on non-speculum clinician-taken samples for cervical screening has not been tried before, therefore novelty is a key study strength. Uptake of the non-speculum sampling approach was reasonable, demonstrating feasibility in a real-world setting. The study also benefited from an ethnically diverse sample that enabled us to examine uptake by ethnicity. This randomised controlled trial was successfully conducted in a deprived and ethnically diverse setting with known capacity issues. There were limited appointments available and long waiting times on telephone booking lines. The study recruited well despite these challenges suggesting it was well designed and conducted.^23^

The main study limitation was the use of GP records to determine participant eligibility and time to conventional screening. GP records are not linked to the English national screening database, therefore records of attendance can be inaccurate. However, the impact of this on the primary endpoint analysis is addressed via randomisation. Potentially, non-speculum clinician sampling uptake was underestimated because of difficulty getting appointments. Similarly, conventional screening uptake in the intervention arm may have been overestimated at practices that provided additional booking systems for the study. The fact that the study documents were only provided in English may have also led to lower uptake of intervention screening tests given the ethnic diversity of the study population.

### Comparison with existing literature

The increased participation of 17% (absolute increase) in the current study is larger than that observed in previous UK self-sampling trials: 6.6% in women aged 50–65 years^24^ and 6–7% for ages 25–65 years.^24^^–^^25^ The observed increased uptake in the current study is also higher than the 10% increased participation associated with self-sampling reported in a rapid review of cancer interventions^26^ and a study of opportunistically offering self-sampling to non-attendees in primary care (9% uptake).^27^ The STRATEGIC trial found no increase in uptake compared with the control arm when young women aged 25 years who had not been screened within 6 months of their first screening invitation were offered the choice of a timed appointment, nurse navigator, or requesting a self-sample kit.^28^ Potentially, the comparatively high uptake is because of the focus on attenders who had lapsed (that is, with the exclusion of never attenders). An alternative explanation is that having both a clinician-taken and self-collected non-speculum sample option enhances uptake synergistically. The observed increase in participation is also substantially larger than that seen in studies using other interventions, such as education^29^ or text message reminders.^30^

Low acceptability of self-sampling and a preference for clinician-taken sampling among Asian women has been reported previously.^31^ High proportions of women from Indian and African Caribbean backgrounds have reported concern about not carrying out the self-sampling test properly.^32^

### Implications for research and practice

Barriers to screening attendance among older women include increased discomfort with the speculum, concerns about body image, musculoskeletal problems with ageing or perceptions of low risk^33^ because of sexual inactivity or long-term monogamy. Offering the choice of self-sampling and non-speculum clinician sampling appears to overcome these barriers and substantially increase uptake. Evidence of the clinical need for non-speculum screening approaches in older women is exemplified by the fact that 3% (7/215) of women aged 50–64 years could not have their routine screening sample taken because of pain (unpublished data by the authors). Having the option to take a non-speculum clinician sample in scenarios where obtaining a speculum sample is difficult would remove the need for further appointments and avoid a potential lapse in screening attendance. This is a benefit that would have an impact for all women of screening age, as difficulty obtaining speculum samples is not limited to older women. Similarly, although never attenders were excluded from the present study, potentially non-speculum clinician sampling has the potential to appeal to those who have been avoiding screening because of the speculum.

The benefit of attending screening (protection offered) increases with longer time since last screen.^34^ Although uptake in this study was lower among women who were ‘very late’ versus those who were ‘late’, the observed 9.5% increase in uptake within 12 months in ‘very late’ women is sufficiently large to confer substantial benefit.

Non-speculum clinician sampling appealed most to women who prefer a clinician to take their sample and are less constrained by practical barriers to getting screened. Conversely, women who find screening embarrassing and have difficulties in getting/making an appointment may prefer self-sampling. A further advantage of non-speculum clinician sampling is that the screener to woman interaction is maintained, which can be a useful platform for enquiry about gynaecological issues and cervical screening.

It appears increasingly likely that offering a choice of test will be important to ensure high uptake.^26^ Non-speculum clinician sampling could be a valuable supplement to self-sampling and warrants further research in larger studies. The rollout of HPV primary testing in many developed countries, including England, makes the introduction of these alternative approaches increasingly feasible. Validation of test performance for this novel approach using paired sampling studies will be important, as will an understanding of the resource and workload implications.
